# Diaqua­bis­(1*H*-imidazole-4-carboxyl­ato-κ^2^ *N* ^3^,*O*)cobalt(II) 

**DOI:** 10.1107/S1600536812037579

**Published:** 2012-09-08

**Authors:** Wen-Sen Chen

**Affiliations:** a School of Chemistry and Environment, South China Normal University, Guangzhou 510006, People’s Republic of China

## Abstract

In the title compound, [Co(C_4_H_3_N_2_O_2_)_2_(H_2_O)_2_], the Co^II^ ion is located on a twofold rotation axis and shows a distorted octa­hedral coordination configuration, defined by two *N*,*O*-bidentate 1*H*-imidazole-4-carboxyl­ate ligands in the equatorial plane and two water mol­ecules in the axial positions. In the crystal, O—H⋯O and N—H⋯O hydrogen bonds link the mol­ecules into a three-dimensional supra­molecular network. π–π stacking inter­actions between the imidazole rings [centroid–centroid distances = 3.4914 (15) and 3.6167 (15) Å] further stabilize the crystal structure.

## Related literature

For related structures, see: Cai *et al.* (2012 ▶); Gryz *et al.* (2007 ▶); Haggag (2005 ▶); Shuai *et al.* (2011 ▶); Starosta & Leciejewicz (2006 ▶); Yin *et al.* (2009 ▶); Zheng *et al.* (2011 ▶).
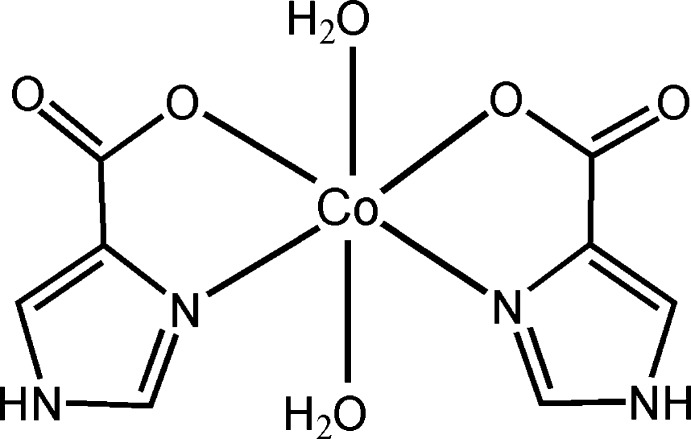


## Experimental

### 

#### Crystal data

[Co(C_4_H_3_N_2_O_2_)_2_(H_2_O)_2_]
*M* *_r_* = 317.13
Orthorhombic, 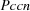

*a* = 7.1216 (18) Å
*b* = 11.780 (3) Å
*c* = 13.536 (3) Å
*V* = 1135.6 (5) Å^3^
*Z* = 4
Mo *K*α radiation
μ = 1.54 mm^−1^
*T* = 298 K
0.35 × 0.33 × 0.30 mm

#### Data collection

Bruker APEXII CCD diffractometer
Absorption correction: multi-scan (*SADABS*; Bruker, 2001 ▶) *T*_min_ = 0.614, *T*_max_ = 0.655
6171 measured reflections
1238 independent reflections
1050 reflections with *I* > 2σ(*I*)
*R*_int_ = 0.024

#### Refinement

*R*[*F* ^2^ > 2σ(*F* ^2^)] = 0.027
*wR*(*F* ^2^) = 0.073
*S* = 1.08
1238 reflections
87 parameters
H-atom parameters constrained
Δρ_max_ = 0.29 e Å^−3^
Δρ_min_ = −0.25 e Å^−3^

### 

Data collection: *APEX2* (Bruker, 2007 ▶); cell refinement: *SAINT* (Bruker, 2007 ▶); data reduction: *SAINT*; program(s) used to solve structure: *SHELXS97* (Sheldrick, 2008 ▶); program(s) used to refine structure: *SHELXL97* (Sheldrick, 2008 ▶); molecular graphics: *ORTEPIII* (Burnett & Johnson, 1996 ▶) and *PLATON* (Spek, 2009 ▶); software used to prepare material for publication: *SHELXL97*.

## Supplementary Material

Crystal structure: contains datablock(s) I, global. DOI: 10.1107/S1600536812037579/hy2579sup1.cif

Structure factors: contains datablock(s) I. DOI: 10.1107/S1600536812037579/hy2579Isup2.hkl

Additional supplementary materials:  crystallographic information; 3D view; checkCIF report

## Figures and Tables

**Table 1:** Hydrogen-bond geometry (Å, °)

*D*—H⋯*A*	*D*—H	H⋯*A*	*D*⋯*A*	*D*—H⋯*A*
O1*W*—H1*WA*⋯O2^i^	0.87	1.99	2.827 (2)	162
O1*W*—H1*WB*⋯O2^ii^	0.86	1.93	2.771 (2)	166
N2—H2⋯O2^iii^	0.86	1.92	2.771 (2)	173

Symmetry codes: (i) 
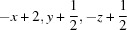
; (ii) 
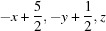
; (iii) 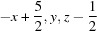.

**Fig. 1.**
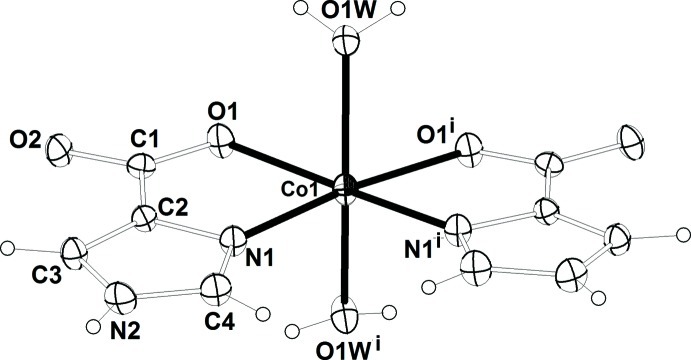
The molecular structure of the title compound, with displacement ellipsoids drawn at the 30% probability level. [Symmetry code: (i) -x+3/2, -y+1/2, z.]

**Fig. 2.**
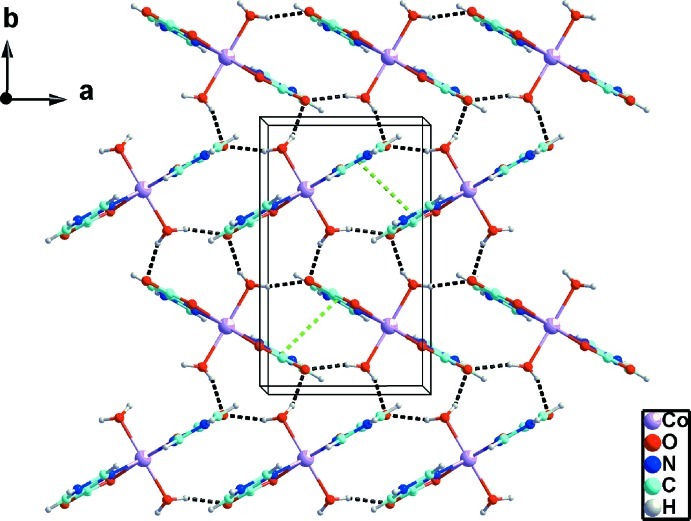
The crystal packing of the title compound, showing the two-dimensional network. Hydrogen bonds and π–π staking interactions are shown as dashed lines.

**Fig. 3.**
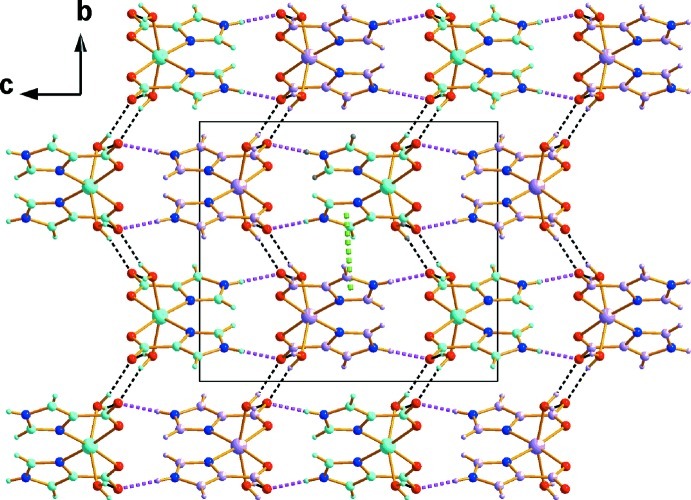
The crystal packing of the title compound, showing the three-dimensional supramolecular network. Hydrogen bonds and π–π staking interactions are shown as dashed lines.

## supplementary crystallographic information

### Comment

There is currently much interest in adopting heterocyclic carboxylic acids as multidentate ligands to prepare new metal coordination polymers. The main reason is that they have versatile coordination behaviors and can form high-dimensional polymers via hydrogen-bonding interactions in the process of self-assembly. 1*H*-Imidazole-4-carboxylic acid (H_2_imc), containing two N atoms of an imidazole group and one carboxylate group, is an excellent candidate for the construction of new coordination polymers. Up to this date, one-, two- and three-dimensional coordination polymers based on the H_2_imc ligand have been documented (Cai *et al.*, 2012; Haggag, 2005; Gryz *et al.*, 2007; Shuai *et al.*, 2011; Starosta & Leciejewicz, 2006; Yin *et al.*, 2009; Zheng *et al.*, 2011). For example, the mononuclear complexes [Cd(Himc)_2_(H_2_O)_2_] and [Zn(Himc)_2_(H_2_O)_2_] have been reported by Yin *et al.* (2009) and Shuai *et al.* (2011), repectively. In this work, we report a Co(II) coordination polymer, [Co(Himc)_2_(H_2_O)_2_], which is isomorphous with the Cd(II) and Zn(II) analogs.

The asymmetric unit of the title compound contains a half of Co^II^ ion, lying on a twofold rotation axis, one Himc anion and one coordinated water molecule. As illustrated in Fig. 1, the Co^II ^ion is six-coordinated by two N and two O atoms from two *cis*-oriented *N,O*-bidentate Himc ligands in the equatorial plane, and two water molecules in the axial positions, forming a slightly distorted octahedral geometry. The Co—N bond length is 2.0786 (16) Å and the Co—O distances are 2.1088 (15) and 2.1793 (14) Å, which are comparable to those of the Cd^II^ and Zn^II^ analogs. In the crystal structure, a pairs of intermolecular O—H···O hydrogen bonds (Table 1) involving the coordinated water (O1W) and the carboxylate O atom (O2) link the molecules into a two-dimensional network in the *ab* plane (Fig. 2). In addition, there exist strong π–π stacking interactions between the imidazole rings in the layer, with a centroid–centroid distatance of 3.4914 (15) Å. These layers are further connected by N—H···O hydrogen bonds (Table 1) involving the imidazole N atom (N2) and the carboxylate O atom (O2), generating a three-dimensional supramolecular network. Another type of π–π stacking interactions with a centroid–centroid distatance of 3.6167 (15) Å also can be observed between the neighbouring layers (Fig. 3).

### Experimental

A mixture of CoCl_2_.6H_2_O (0.20 mmol), H_2_imc (0.20 mmol) and 6 ml EtOH/H_2_O (v/v 1:1) was sealed into a 10 ml sample bottle reactor and heated at 373 K for 48 h under autogenous pressure, and then slowly cooled to room temperature at a rate of 2 K/h. Red block crystals of the title compound were isolated, washed with distilled water, and dried in air (yield: 45%).

### Refinement

C- and N-bound H atoms were positioned geometrically and refined using a riding model, with C—H = 0.93 and N—H = 0.86 Å and with *U*_iso_(H) = 1.2*U*_eq_(C, N). H atoms of the water molecule were located from a difference Fourier map and refined as riding, with O—H bond lenghts restrained to 0.86 Å.

### Crystal data

**Table:**

[Co(C_4_H_3_N_2_O_2_)_2_(H_2_O)_2_]	*F*(000) = 644
*M**_r_* = 317.13	*D*_x_ = 1.855 Mg m^−^^3^
Orthorhombic, *P**c**c**n*	Mo *K*α radiation, λ = 0.71073 Å
Hall symbol: -P 2ab 2ac	Cell parameters from 2075 reflections
*a* = 7.1216 (18) Å	θ = 3.0–27.3°
*b* = 11.780 (3) Å	µ = 1.54 mm^−^^1^
*c* = 13.536 (3) Å	*T* = 298 K
*V* = 1135.6 (5) Å^3^	Block, red
*Z* = 4	0.35 × 0.33 × 0.30 mm

### Data collection

**Table:**

Bruker APEXII CCD diffractometer	1238 independent reflections
Radiation source: fine-focus sealed tube	1050 reflections with *I* > 2σ(*I*)
Graphite monochromator	*R*_int_ = 0.024
φ and ω scans	θ_max_ = 27.0°, θ_min_ = 3.0°
Absorption correction: multi-scan (*SADABS*; Bruker, 2001)	*h* = −9→9
*T*_min_ = 0.614, *T*_max_ = 0.655	*k* = −12→15
6171 measured reflections	*l* = −17→16

### Refinement

**Table:**

Refinement on *F*^2^	Primary atom site location: structure-invariant direct methods
Least-squares matrix: full	Secondary atom site location: difference Fourier map
*R*[*F*^2^ > 2σ(*F*^2^)] = 0.027	Hydrogen site location: inferred from neighbouring sites
*wR*(*F*^2^) = 0.073	H-atom parameters constrained
*S* = 1.08	*w* = 1/[σ^2^(*F*_o_^2^) + (0.0317*P*)^2^ + 0.7299*P*] where *P* = (*F*_o_^2^ + 2*F*_c_^2^)/3
1238 reflections	(Δ/σ)_max_ < 0.001
87 parameters	Δρ_max_ = 0.29 e Å^−^^3^
0 restraints	Δρ_min_ = −0.25 e Å^−^^3^

### Special details

**Table:**

Geometry. All esds (except the esd in the dihedral angle between two l.s. planes) are estimated using the full covariance matrix. The cell esds are taken into account individually in the estimation of esds in distances, angles and torsion angles; correlations between esds in cell parameters are only used when they are defined by crystal symmetry. An approximate (isotropic) treatment of cell esds is used for estimating esds involving l.s. planes.
Refinement. Refinement of F^2^ against ALL reflections. The weighted R-factor wR and goodness of fit S are based on F^2^, conventional R-factors R are based on F, with F set to zero for negative F^2^. The threshold expression of F^2^ > 2sigma(F^2^) is used only for calculating R-factors(gt) etc. and is not relevant to the choice of reflections for refinement. R-factors based on F^2^ are statistically about twice as large as those based on F, and R- factors based on ALL data will be even larger.

### Fractional atomic coordinates and isotropic or equivalent isotropic displacement parameters (Å^2^)

**Table:**

	*x*	*y*	*z*	*U*_iso_*/*U*_eq_	
Co1	0.7500	0.2500	0.13193 (2)	0.02397 (14)	
O1	0.95746 (19)	0.17187 (13)	0.22880 (10)	0.0305 (3)	
O1W	0.8973 (2)	0.40251 (13)	0.15738 (11)	0.0351 (4)	
O2	1.23719 (18)	0.08486 (12)	0.22678 (10)	0.0302 (3)	
N1	0.9449 (2)	0.18857 (14)	0.03090 (11)	0.0270 (4)	
C3	1.2150 (3)	0.09661 (18)	0.00537 (15)	0.0304 (4)	
H3	1.3262	0.0569	0.0155	0.036*	
C1	1.0961 (3)	0.12982 (16)	0.18488 (14)	0.0243 (4)	
C2	1.0934 (3)	0.13421 (16)	0.07551 (14)	0.0235 (4)	
C4	0.9791 (3)	0.18410 (18)	−0.06466 (14)	0.0314 (5)	
H4	0.9018	0.2150	−0.1131	0.038*	
N2	1.1403 (2)	0.12911 (15)	−0.08276 (12)	0.0327 (4)	
H2	1.1882	0.1166	−0.1401	0.039*	
H1WA	0.8364	0.4481	0.1962	0.039*	
H1WB	1.0072	0.3950	0.1833	0.039*	

### Atomic displacement parameters (Å^2^)

**Table:**

	*U*^11^	*U*^22^	*U*^33^	*U*^12^	*U*^13^	*U*^23^
Co1	0.0206 (2)	0.0304 (2)	0.0209 (2)	0.00483 (15)	0.000	0.000
O1	0.0258 (8)	0.0427 (8)	0.0230 (7)	0.0060 (6)	0.0011 (6)	−0.0005 (6)
O1W	0.0241 (8)	0.0367 (8)	0.0445 (9)	0.0039 (6)	−0.0032 (6)	−0.0095 (7)
O2	0.0225 (7)	0.0413 (8)	0.0267 (7)	0.0027 (6)	−0.0034 (6)	0.0047 (6)
N1	0.0252 (9)	0.0331 (9)	0.0226 (8)	0.0046 (7)	0.0017 (6)	0.0016 (7)
C3	0.0258 (10)	0.0356 (11)	0.0297 (10)	0.0038 (8)	0.0005 (8)	−0.0026 (9)
C1	0.0233 (10)	0.0246 (9)	0.0251 (9)	−0.0032 (7)	−0.0019 (8)	0.0010 (8)
C2	0.0218 (10)	0.0251 (9)	0.0237 (9)	0.0005 (7)	−0.0002 (7)	0.0007 (7)
C4	0.0303 (11)	0.0408 (12)	0.0230 (10)	0.0041 (9)	0.0004 (8)	0.0031 (9)
N2	0.0327 (10)	0.0424 (10)	0.0231 (8)	0.0023 (8)	0.0073 (7)	−0.0015 (7)

### Geometric parameters (Å, º)

**Table:**

Co1—N1	2.0786 (16)	N1—C2	1.376 (2)
Co1—O1W	2.1088 (15)	C3—C2	1.359 (3)
Co1—O1	2.1793 (14)	C3—N2	1.361 (3)
O1—C1	1.254 (2)	C3—H3	0.9300
O1W—H1WA	0.87	C1—C2	1.482 (3)
O1W—H1WB	0.86	C4—N2	1.341 (3)
O2—C1	1.269 (2)	C4—H4	0.9300
N1—C4	1.317 (2)	N2—H2	0.8600
			
N1^i^—Co1—N1	97.72 (9)	C4—N1—C2	105.69 (16)
N1^i^—Co1—O1W	98.23 (6)	C4—N1—Co1	141.57 (14)
N1—Co1—O1W	94.12 (6)	C2—N1—Co1	112.73 (12)
N1^i^—Co1—O1W^i^	94.12 (6)	C2—C3—N2	105.76 (18)
N1—Co1—O1W^i^	98.23 (6)	C2—C3—H3	127.1
O1W—Co1—O1W^i^	161.19 (9)	N2—C3—H3	127.1
N1^i^—Co1—O1	174.63 (6)	O1—C1—O2	125.17 (18)
N1—Co1—O1	78.23 (6)	O1—C1—C2	116.70 (16)
O1W—Co1—O1	85.65 (6)	O2—C1—C2	118.13 (17)
O1W^i^—Co1—O1	83.06 (6)	C3—C2—N1	109.55 (17)
N1^i^—Co1—O1^i^	78.23 (6)	C3—C2—C1	132.70 (18)
N1—Co1—O1^i^	174.63 (6)	N1—C2—C1	117.72 (16)
O1W—Co1—O1^i^	83.06 (6)	N1—C4—N2	110.91 (18)
O1W^i^—Co1—O1^i^	85.65 (6)	N1—C4—H4	124.5
O1—Co1—O1^i^	106.02 (7)	N2—C4—H4	124.5
C1—O1—Co1	114.54 (12)	C4—N2—C3	108.07 (17)
Co1—O1W—H1WA	112.2	C4—N2—H2	126.0
Co1—O1W—H1WB	115.5	C3—N2—H2	126.0
H1WA—O1W—H1WB	105.7		
			
N1—Co1—O1—C1	1.74 (14)	N2—C3—C2—N1	0.4 (2)
O1W—Co1—O1—C1	−93.39 (14)	N2—C3—C2—C1	−177.63 (19)
O1W^i^—Co1—O1—C1	101.68 (14)	C4—N1—C2—C3	−0.5 (2)
O1^i^—Co1—O1—C1	−174.89 (15)	Co1—N1—C2—C3	−179.88 (13)
N1^i^—Co1—N1—C4	4.6 (2)	C4—N1—C2—C1	177.83 (17)
O1W—Co1—N1—C4	−94.3 (2)	Co1—N1—C2—C1	−1.5 (2)
O1W^i^—Co1—N1—C4	99.9 (2)	O1—C1—C2—C3	−179.0 (2)
O1—Co1—N1—C4	−179.0 (2)	O2—C1—C2—C3	1.4 (3)
N1^i^—Co1—N1—C2	−176.42 (16)	O1—C1—C2—N1	3.1 (2)
O1W—Co1—N1—C2	84.68 (14)	O2—C1—C2—N1	−176.53 (17)
O1W^i^—Co1—N1—C2	−81.10 (14)	C2—N1—C4—N2	0.5 (2)
O1—Co1—N1—C2	0.00 (13)	Co1—N1—C4—N2	179.49 (16)
Co1—O1—C1—O2	176.63 (15)	N1—C4—N2—C3	−0.2 (2)
Co1—O1—C1—C2	−3.0 (2)	C2—C3—N2—C4	−0.1 (2)

Symmetry code: (i) −*x*+3/2, −*y*+1/2, *z*.

### Hydrogen-bond geometry (Å, º)

**Table:**

*D*—H···*A*	*D*—H	H···*A*	*D*···*A*	*D*—H···*A*
O1*W*—H1*WA*···O2^ii^	0.87	1.99	2.827 (2)	162
O1*W*—H1*WB*···O2^iii^	0.86	1.93	2.771 (2)	166
N2—H2···O2^iv^	0.86	1.92	2.771 (2)	173

Symmetry codes: (ii) −*x*+2, *y*+1/2, −*z*+1/2; (iii) −*x*+5/2, −*y*+1/2, *z*; (iv) −*x*+5/2, *y*, *z*−1/2.
